# Identification of a Human Cyclin D1-Derived Peptide that Induces Human Cytotoxic CD4 T Cells

**DOI:** 10.1371/journal.pone.0006730

**Published:** 2009-08-25

**Authors:** Tao Dao, Tatyana Korontsvit, Victoria Zakhaleva, Kurtis Haro, Jonathan Packin, David A. Scheinberg

**Affiliations:** Molecular Pharmacology and Chemistry Program, Memorial Sloan Kettering Cancer Center, New York, New York, United States of America; New York University School of Medicine, United States of America

## Abstract

Cyclin D1 is over-expressed in various human tumors and therefore can be a potential oncogenic target antigen. However, only a limited number of T cell epitopes has been characterized. We aimed at identifying human cyclin D1-derived peptides that include both CD4 and CD8 T cell epitopes and to test if such multi-epitope peptides could yield improved cytotoxic CD8 T cell responses as well as cytotoxic CD4 T cells. Five HLA-DR.B1-binding peptides containing multiple overlapping CD4 epitopes and HLA-A0201-restricted CD8 T cell epitopes were predicted by computer algorithms. Immunogenicity of the synthetic peptides was assessed by stimulating T cells from healthy donors *in vitro* and the epitope recognition was measured by IFN-γ ELISPOT and ^51^Chromium release assays. A HLA-DR.B1 peptide, designed “DR-1”, in which a HLA-A0201-binding epitopes (D1-1) was imbedded, induced CD3 T cell responses against both DR-1 and D1-1 peptides in IFN-γ ELISPOT assay. This suggested processing of the shorter D1-1 epitope from the DR-1 sequence. However, only DR-1-stimulated CD4 or CD3 T cells possessed cytotoxicity against peptide-pulsed autologous DCs and a cancer cell line, that expresses a high level of cyclin D1. Monoclonal antibody to HLA-DR abrogated the epitope-specific responses of both CD3 and CD4 T cells, demonstrating class II-mediated killing. Our studies suggest a possible role of CD4 T cells in anti-tumor immunity as cytotoxic effectors against HLA-DR expressing cancers and provide a rationale for designing peptide vaccines that include CD4 epitopes.

## Introduction

Cyclin D1 is a key regulatory protein during the progression of cell cycle through G1 phase. Growth factor stimulation triggers an increase in cyclin D1 transcript and translation as well as its assembly into an active cyclin D1/cyclin-dependent kinase (CDK) 4 complex. The active kinase triggers phosphorylation of the retinoblastoma protein (RB), relieving its transcriptional repressive activities and its capacity to regulate components of the DNA replication and G2/M progression [1], [2]. It is normally expressed at low levels in some organs and tissues, but is over-expressed in a number of human cancers, including mantle cell lymphoma (MCL), breast cancer, esophageal cancer and non-small cell lung cancer (NSCLC) [3], [4], [5], [6]. Recent evidence demonstrated the presence of an alternatively spliced cyclin D1 transcript, called cyclin D1b. This variant transcript showed a failure of splicing at the 3′ end of exon 4 and as a result, the expected protein product is altered at its C-terminus. Unlike wild type (WT) cyclin D1a, cyclin D1b is retained in the nuclus through the cell cycle where its constitutive expression promotes oncogenic transformation. Studies from esophageal cancer and murine model of MCL have suggested that this mutant protein is oncogenic [7], [8], [9].

These studies suggest that cyclin D1 could be a potential new target in a wide range of cancers. However, the non-enzymatic cyclin D1 molecule does not fall into a class of proteins that are conventionally considered to be targetable by small molecule inhibitors. In addition, cyclin D1 is an intracellular protein, and thus a specific antibody (Ab) cannot be directly bound to the protein. Therefore, generating cytotoxic T lymphocytes (CTLs) that kill cyclin D1-expressing tumor cells could be a promising approach. However, there have been limited reports identifying T cell eitopes derived from cyclin D1 and its potential use for immunotherapy. Stauss et al. have first reported a HLA-A0201-binding peptide (cyclin D1 101–109) that induce allogeneic CTLs in human *in vitro* [10]. Such allogeneic CTL would be useful for adoptive immunotherapy involving bone marrow transplant from partially MHC-mismatched donors. However, active immunotherapy in other settings require the peptide vaccines be presented by host antigen presenting cells (APCs) to T cells to generate anti-tumor immune responses. Recently, Kondo et al. have reported that two cyclin D1-derived peptides, including the one from Stauss group, could induce autologous CD8 CTLs in both healthy donors and cancer patients [11].

Induction of strong CD8 CTL responses has long been the goal of most peptide vaccine strategies [12], [13]. However, this approach has achieved limited success in clinical trials [14], [15]. A strategy to include CD4 T cell epitopes in vaccine design, aimed at the helper function of CD4 T cells in generation of long-lasting memory CD8 T cells, has received promising results [16]. However, the role of CD4 T cells in directly killing tumor cells or clearing tumors, independent of CD8 T cells, is less well-studied.

In the present study, we set out to address the questions of whether including CD4 epitopes enhances CD8 T cell responses and whether CD4 T cells can be cytotoxic to cancer cells. Our approach was to design peptides derived from human cyclin-D1-a protein, that 1) included both CD4 and CD8 epitopes in the same sequence; 2) could result in processing and presentation of CD8 epitopes; 3) could potentially be presented by multiple class II molecules, rather than being limited to a single DR type. Due to the permissive nature of the HLA class II binding pocket, this latter criteria was possible. Such peptides would therefore be capable of priming T cells from a larger percentage of the target population, allowing a vaccine strategy that would address a large segment of the population.

## Materials and Methods

### Synthetic peptides

All peptides were purchased and synthesized by Genemed Synthesis, Inc. (San Antonio, TX). Peptides were sterile and 70% to 90% pure. The peptides were dissolved in DMSO and diluted in saline at 5 mg/mL and stored at −80°C. Amino acid sequences and predicted binding of putative CD4+epitopes to HLA-DRB1 molecules and HLA-A0201 were identified using the predictive algorithm of the SYFPEITHI database 3 (http://www.syfpeithi.de/) and RANKPEP (http://bio.dfci.harvard.edu/Tools/rankpep.html). A CD4 specificity control peptide used for HLA-DR was the JAK-2-derived DR.B1-binding peptide JAK2-DR (GVCVCGDENILVQEF, SYFPEITHI score 24–17; unpublished data). This peptide induces a CD4 response after multiple stimulations. A CD8 specificity control peptide used for HLA-A0201 was the ewing sarcoma-derived peptide EW (QLQNPSYDK, RANKPEP score 29; unpublished data).

### Cells lines, cytokines and antibodies

The human breast cancer cell line MDA-MB-231 (ATCC number HTB-26) was used as a target for measuring cytotoxicity of T cells [10]. The cell line was pre-treated with human recombinant IFN-γ (100 ng/ml) for two days to up-regulate HLA-DR expression. The cells were washed thoroughly before being used as targets for IFN-γ ELISPOT and ^51^Cr-release assays. The haplotype of the cells are: HLA-A0201/0217, B4002/4101, C0202/1701, DR.B1*0701/1305 and DQ.B1*0202/0301. Human granulocyte-macrophage colony-stimulating factor (GM-CSF), interleukin (IL)-1β, IL-4, IL-6, IL-15, tumor necrosis factor (TNF)-α and prostglandin E2 (PGE2) were purchased from R&D Systems (Minneapolis, MN). Beta 2-microglobulin (β2-m) and human IFN-γ were purchased from Sigma (St. Louis, MO). The antibodies used for immunofluorescence assays including mAbs to human CD3, CD4, CD8, CD56, CD14, CD19, CD83, CD86, CD11C, HLA-DR and isotype controls were obtained from BD Biosciences (San Diego, CA). Neutralizing Abs against HLA-A (clone W6/32), HLA-A2 (clone BB7), HLA-DR (clone L243) and isotype controls mouse IgG2a (clone F23.2: anti-human Vβ8 and clone KJ1-26: anti-mouse TCR DO-11) were obtained from the Monoclonal Antibody Facility at MSKCC (New York, NY). Anti-human cyclin D1 mAb (clone 5D4) was purchased from Beckman Coulter (Fullerton, CA). Goat F (ab') 2 anti-mouse Ig's conjugated to fluorescent was purchased from Biosource (Camarill, CA). Cell isolation kits for CD3, CD4, CD8 and CD25 were purchased from Miltenyi Biotec. (Bergisch Gladbach, Germany).

### Flow cytometry and phenotypic analysis

Cyclin D1 expression was measured by intracellular protein staining using mAb to human cyclin D1 (clone 5D4) and Cytofix/CytoPerm kit (BD Biosciences), according to the instructions of the manufacturer. For cell surface staining, cells were incubated with appropriate mAbs for 30 minutes on ice, washed, and incubated with secondary antibody reagents when necessary. Analysis was done on a BD Biosciences FACScan (San Jose, CA) or LSR (Mountain View, CA).

### *In vitro* stimulation and human T-cell cultures

After informed consent on Memorial Sloan-Kettering Cancer Center Institutional Review Board approved protocols, peripheral blood mononuclear cells (PBMC) from HLA-typed healthy donors were obtained by Ficoll density centrifugation. CD14+ monocytes were isolated by positive selection using mAb to human CD14 coupled with magnetic beads (Miltenyi Biotec) and were used for the first stimulation of T cells. The CD14- fraction of PBMC were used for isolation of CD3, by negative immunomagnetic cell separation using a pan T cell isolation kit (Miltenyi Biotec). The purity of the cells was always more than 98%. T cells were stimulated for 7 days in the presence of RPMI 1640 supplemented with 5% autologous plasma (AP), 20 ug/mL synthetic peptides, 1 ug/mL β2-m, and 10 ng/mL IL-15. Monocyte-derived dendritic cells (DCs) were generated from CD14+ cells, by culturing the cells in RPMI 1640 medium supplemented with 1% AP, 500 units/mL recombinant IL-4, and 1,000 units/mL GM-CSF. On days 2 and 4 of incubation, fresh medium with IL-4 and GM-CSF was either added or replaced half of the culture medium. On day 5, 20 ug/mL class II peptide was added to the immature DCs. On day 6, maturation cytokine cocktail was added (IL-4, GM-CSF, 500 IU/mL IL-1, 1,000 IU/mL IL-6, 10 ng/ml TNF-α, and 1 ug/mL PGE-2). On day 7 or 8, T cells were re-stimulated with mature DCs at a 30∶1, T: APC ratio, with IL-15. In most cases, T cells were stimulated 3 times in the same manner, using either DCs or CD14+ cells as antigen-presenting cells (APCs). A week after final stimulation, the peptide-specific T cell response was examined by IFN-γ enzyme-linked immunospot (ELISPOT) assay and the cytotoxicity was tested, by ^51^chromium (Cr)-release assay. To deplete the T-reg cell population, we first depleted CD25+ T cells from CD14- fraction of PBMC, using an mAb to human CD25 coupled with magnetic beads (Miltenyi Biotec). The CD25-fraction was subjected to further purification for either CD3+ or CD4+ fraction. CD3 T cell population was isolated by negative selection as described above, and CD4 T cell population was isolated by positive selection using mAb to human CD4-magnetic beads. This yielded two final populations: CD3+CD25- or CD4+CD25- T cells.

### IFN-γ ELISPOT

HA-Multiscreen plates (Millipore) were coated with 100 uL of mouse anti-human IFN-γ antibody (10 Ag/mL; clone 1-D1K; Mabtech) in PBS, incubated overnight at 4°C, washed with PBS to remove unbound antibody, and blocked with RPMI 1640/10% autologous plasma (AP) for 2 h at 37°C. Purified CD4+, CD8+, or CD3+ T cells (>98% pure) were plated with either autologous CD14+ (10∶1 E: APC ratio) or autologous DCs (30∶1 E: APC ratio). Various test peptides were added to the wells at 20 ug/mL. Negative control wells contained APCs and T cells without peptides or with irrelevant peptides. Phytohemagglutinin (PHA, Sigma) at a concentration of 20 ug/ml was used as a positive control for the assay. However, the PHA data was not shown in the Figs, because PHA induces such a large amount of spots that it is not possible to plot in the same Figure with the peptide-induced spots. All conditions were done in triplicate. Blocking experiments were done in which 50 ug/mL of blocking mAbs or irrelevant control mAbs were pre-incubated with 10^6^ CD14+ or other target cells for 30 minutes at 37°C and then were presented in the cultures throughout the incubation period of time. Microtiter plates were incubated for 20 h at 37°C and then extensively washed with PBS/0.05% Tween and 100 ul/well biotinylated detection antibody against human IFN-γ (2 ug/mL; clone 7-B6-1; Mabtech) was added. Plates were incubated for an additional 2 h at 37°C and spot development was done as described [16]. Spot numbers were automatically determined with the use of a computer-assisted video image analyzer with KS ELISPOT 4.0 software (Carl Zeiss Vision).

### ^51^Chromium release assay

The presence of specific CTLs was measured in a standard 4-hour chromium release assay as described [17]. Briefly, target cells alone, or pulsed with 20 ug/mL of synthetic peptides for 2 hours (in some cases for over night) at 37°C, are labeled with 50 uCi/million cells of Na2 51CrO4 (NEN Life Science Products, Inc.). After extensive washing, target cells are incubated with T cells at E: T ratios ranging from 100∶1 to 10∶1. All conditions were done in triplicate. Plates were incubated for 4–5 hrs at 37°C in 5% CO2. Supernatant fluids were harvested and radioactivity was measured in a gamma counter. Percentage specific lysis was determined from the following formula: [(experimental release−spontaneous release)/(maximum release−spontaneous release)]×100%. Maximum release was determined by lysis of radiolabeled targets in 1% SDS.

## Results

### Identification of cyclin D1-derived peptides with high predicted binding to multiple class II HLA-DR.B1 and class I HLA-A0201 molecules

Physical linking of T helper and CTL epitopes increases the magnitude and duration of the CTL response, suggesting that the presentation of both T helper and CTL epitopes on a single antigen presenting cell is more efficient than when two epitopes are presented on different APCs [18]. CD4 T cells help CD8 CTL by fully activating DCs through the CD40/CD40L signaling as well as by producing IL-2 and IFN-γ [19], [20], [21]. Therefore, a peptide combining both CD4 and CD8 epitopes could be advantageous over the single class I epitope in eliciting effective immune response for vaccine design. In addition, designing a class II peptide that contains a class I epitope would allow us to assess the processing and presentation of the short epitope, assuring that such a peptide would be a valid vaccine candidate. Because we recognize that prediction of immunogenic antigens results in both false negative and false positive sequences, our conclusions would have to rely ultimately on tests for T cell recognition of the sequences *in vitro*. Our approach included the following steps: A) Prediction of potential epitopes by computer algorithms. This would serve to limit the number of possible sequence choices; B) Stimulation of T cells with selected peptides to confirm the immunogenicity of the epitopes; C) Testing of the response of the peptide-stimulated T cells against tumor cells that highly express cyclin D1, to confirm the processing and the presentation of the epitopes.

We first screened human cyclin D1-a protein sequences using the BIMAS, SYFPEITHI and RANKPEP algorithms for binding to HLA-A0201; and a number of peptides were identified that had predicted high-affinity binding to HLA-A0201. Among them, the CD8 epitopes with RANKPEP- predicted C-terminal proteasome cleavage, were selected.

In parallel, the cyclin D1 sequences containing these HLA-A0201 epitopes but extended for several amino acids in both directions are further screened in silico for their predicted binding efficiency to multiple HLA-DR.B1 molecules that are highly expressed in the Caucasian population. Since the SYFPEITHI program only predicts binding of 15 amino acid sequences to HLA-DR molecules, we designed longer peptides by adding flanking residues in both ends that resulted in overlapping multiple epitopes for CD4 (15 amino acids) within one sequence. We designed five HLA-DR.B1-binding peptides; each contains multiple epitopes for HLA-DR.B1 molecules and one or two epitopes for HLA-A0201 molecules. The peptide sequences and their binding prediction for HLA-DR.B1 (SYFPEITHI) and A0201 molecules (RANKPEP) are shown (Table 1). The putative HLA-DR.B1-binding peptides are designed as DR-1, 2, 3, 4 and 5. The putative HLA-A0201-binding peptides are named as D1-1, -2, -3, -4, -5, -6, -7 and 8. As an example, DR-1 peptide (26 amino acids) spanning cyclin D1-a amino acids 42–67, is predicted to generate multiple overlapping 15 amino acid epitopes that have a wide range of binding efficiency to HLA-DR.B1-0101, -0301, -0401, -0701, 1105 & 1501. An example of two potential 15 amino acid HLA-DR.B1-binding epitopes within DR-1 peptide is shown in Table 1. The epitope vsyfkcvqkevlpsm (position 42–56) binds to multiple HLA-DR.B1 subtypes with scores ranging from 25 to12 out of 35 maximum score. Similarly, the epitope lpsmrkivatwmlev (position 53–67) shows scores that range of 35–11 binding to multiple HLA-DR.B1 molecules. There are additional multiple 15 mer fragments that were predicted to be potential epitopes for HLA-DR.B1 molecule by SYFPEITHI algorithm as well (data not shown). In addition, two HLA-A0201-binding, 9 mer peptides are embedded within the DR-1 peptide; the D1-1 peptide spanning sequences 59–67 and the D1-2 spanning sequences 52–60, displayed binding scores of 85 and 59, respectively, by RANKPEP prediction. Importantly, the HLA-A0201-binding peptides are predictedto be cleaved at their C-terminals, suggesting a possibility of being processed by the proteosome. Such multi-epitope peptides should offer an advantage of inducing simultaneously robust CD4+ and CD8+ T-cell responses.

**Table 1:** Peptides from human cyclin D1-a that are predicted to bind to HLA-DR.B1 and embedded short peptides (in italics) that bind to HLA-A0201 molecules.

Name	Sequences	Position	Score*
**DR-1**	VSYFKCVQKEVLPSMRKIVATWMLEV	42–67	
	VSYFKCVQKEVLPSM	42–56	*HLA-DR.B1: 25–12
	LPSMRKIVATWMLEV	53–67	HLA-DR.B1: 35–11
***D1-1***	*IVATWMLEV*	59–67	*HLA-A0201: 80
***D1-2***	*VLPSMRKIV*	52–60	HLA-A0201: 67
**DR-2**	AQTFVALCATDVKFISNPPSMVAAGSVV	182–209	
	AQTFVALCATDVKFI	182–196	HLA-DR.B1: 36–16
	ATDVKFISNPPSMVA	190–204	HLA-DR.B1: 26–18
***D1-3***	*FVALCATDV*	185–193	HLA-A0201: 61
***D1-4***	*SMVAAGSVV*	201–209	HLA-A0201: 75
**DR-3**	LLLVNKLKWNLAAMT	142–156	HLA-DR.B1: 36–20
***D1-7***	*LVNKLKWNL*	144–152	HLA-A0201: 48
**DR-4**	MNYLDRFLSLEPVKK	82–96	HLA-DR.B1: 34–12
***D1-5***	*NYLDRFLSL*	83–91	HLA-A0201: 46
**DR-5**	VQGLNLRSPNNFLSYYR	212–228	
	VQGLNLRSPNNFLSY	212–226	HLA-DR.B1: 24–3
	GLNLRSPNNFLSYYR	214–228	HLA-DR.B1: 24–13
***D1-6***	*NLRSPNNFL*	216–224	HLA-A0201: 51
***D1-8***	*SPNNFLSY*	219–226	HLA-A0201: 28

* SYFPEITHI prediction software was used for HLA-DR.B1-binding prediction. Each epitope has different binding affinity to various DR.B1 subtypes, which was summarized as the range of binding scores. RANKPEP prediction software was used for HLA-A0201 prediction and C-terminal cleavage.

### Multiple cyclin D1-derived peptides induced peptide-specific CD3 T cell responses

**Figure 1:**
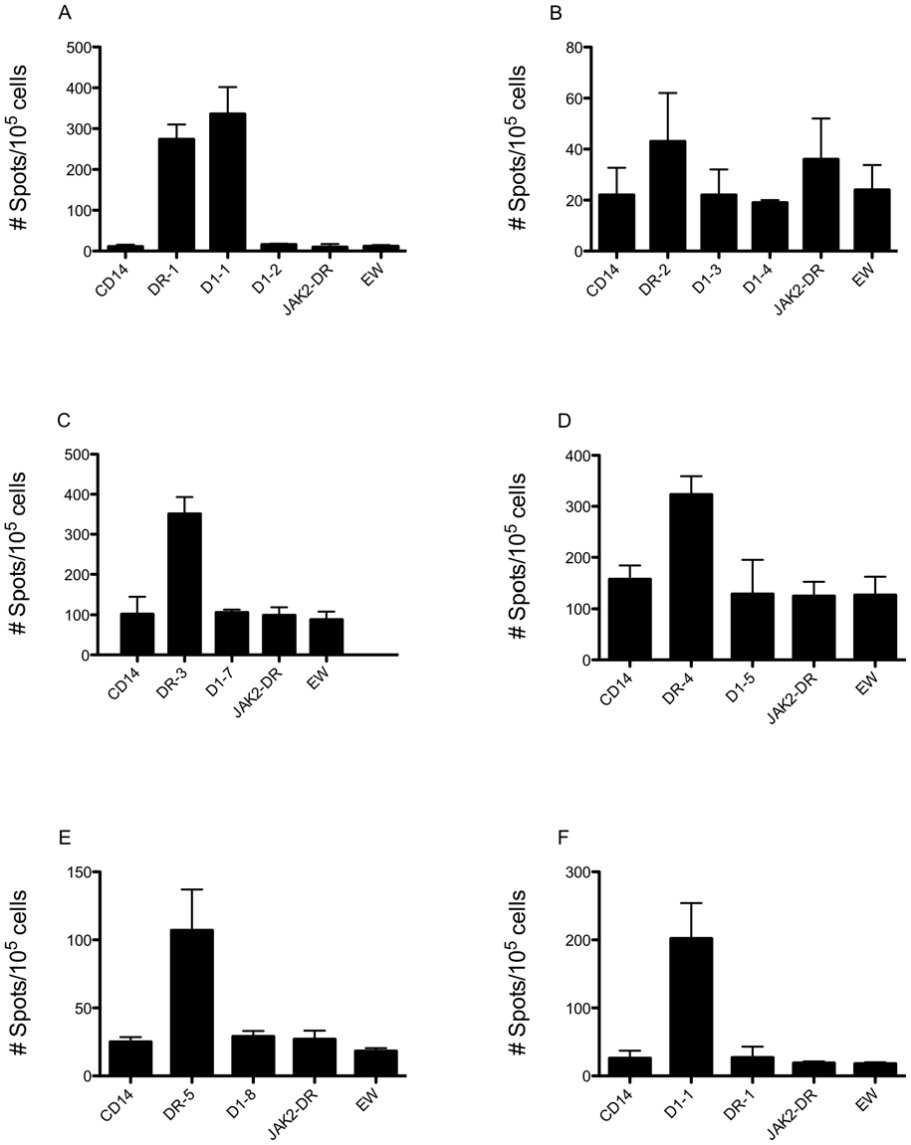
IFN-γ ELISPOT by CD3 T cells stimulated with cyclin D1 peptides. CD3 T cells from healthy donors with HLA-A0201 and various HLA-DR.B1 haplotypes were stimulated three times with human cyclin D1-derived peptides DR-1 (A), DR-2 (B), DR-3 (C), DR-4 (D), DR-5 (E) or D1-1 (F), as described in the Materials and Methods. Stimulated CD3 T cells were challenged in IFN-γ ELISPOT assay with the stimulating HLA-DR.B1 class II peptides and the HLA-A0201 class I peptides that were imbedded within the class II peptides, as indicated. Controls are: un-pulsed CD14+ cells, irrelevant peptides JAK2-DR for HLA-DR.B1 and EW for HLA-A0201. The results represent the average spots in triplicate cultures plus/minus standard deviation (SD). The figures show one representative experiment from multiple similar experiments.

Web-based predictive algorithms are only 70% reliable in identifying peptide epitopes capable of stimulating T cells [22]. Therefore, *in vitro* stimulation assays are necessary to confirm those peptides that can stimulate T cells. CD3 T cells from healthy donors were initially used as effectors because they could respond to both class I and II peptides. The cells were stimulated with peptide-loaded CD14+ positive cells or autologous DCs for three rounds and the IFN-γ secretion was measured in ELISPOT assay. All of the HLA-DR.B1 peptides, except for DR-2, could induce peptide-specific CD4+ responses (Fig. 1A–E). However, DR-1 consistently induced the best response in a variety of HLA-DR.B1 settings, reproduced multiple times in more than 10 healthy donors, each with different HLADR.B1 haplotypes.

More importantly, CD3 T cells stimulated with DR-1 peptide also reacted to HLA-A0201-restricted class I peptide D1-1. This suggested that the D1-1 peptide might be processed and presented to CD8 T cells. The other HLA-A0201-restricted peptides imbedded within DR-1 (D1-2), DR-2 (D1-3 and D1-4), DR-3 (D1-7), DR-4 (D1-5) and DR-5 (D1-6 and D1-8) were not reactive with the CD3 T cells stimulated with their respective long peptides, although the epitopes were predicted to be processed in C-terminus. Since the DR-1 peptide offered the possibility of inducing simultaneously a robust CD4+ and CD8+ T-cell response against DR-1 and D1-1 peptides, we were interested in further pursuing this peptide as a potential vaccine candidate. To confirm that the D1-1 peptide was immunogenic alone, CD3 T cells were stimulated with D1-1 peptide and it induced strong peptide-specific IFN-γ secretion (Fig. 1F).

### Cyclin D1-DR-1 peptide induced CD4 T cell response in healthy donors with multiple HLA-DR.B1 haplotypes

**Figure 2:**
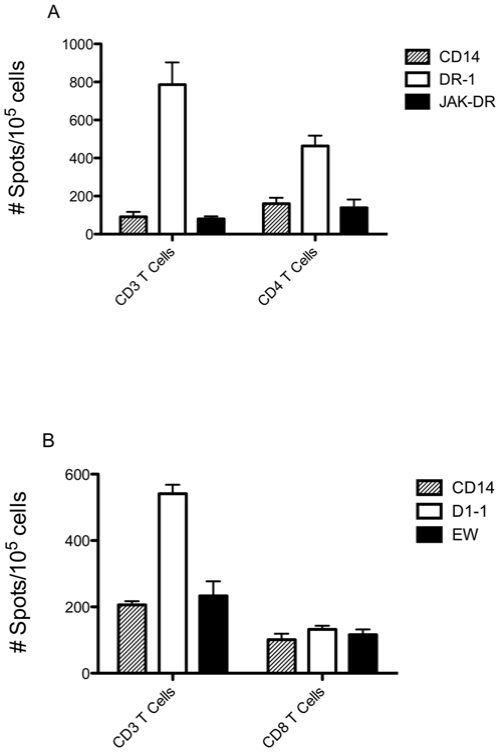
IFN-γ ELISPOT by CD3, CD4 or CD8 T cells stimulated with cyclin D1-DR1 or D1-1 peptides. (A) CD3 or CD4 T cells from a healthy donor (HLA-A0201 and DR.B1 0104/1104) were stimulated twice with human cyclin D1-derived peptides DR-1, as described in the Materials and Methods. (B) CD3 or CD8 T cells from a healthy donor (HLA-A0201 and HLA-DR.B1 0801/0901) were stimulated three times with human cyclin D1-derived peptides D1-1. Stimulated T cells were challenged in IFN-γ ELISPOT assay with the stimulating peptide DR-1 or D1-1. The controls are: un-pulsed CD14+ cell as APCs, irrelevant peptides JAK2-DR for HLA-DR.B1 and EW for HLA-A0201. The results represent the average spots in triplicate cultures +/− SD. The figures show one representative data from multiple similar experiments.

Using CD3 T cells as effectors allowed us to simultaneously measure both class II and class I-restricted T cell responses, if the class I peptides were processed from the longer class II sequences. In addition, the presence of CD4 T cells should improve CD8 T cell response, as demonstrated before in other systems [23], [24], [25], [26]. To further clarify the role of CD4 and CD8 T cells in the mixed CD3 T cell responses specific for DR-1 and D1-1 peptides, we next purified CD3, CD4 and CD8 T cell populations from healthy donors (HLA-A0201 positive with various HLA-DR.B1 haplotypes) and compared their responses against the stimulating peptides. When CD3 and CD4 T cells from the same donor were stimulated with the DR-1 peptide, CD3 T cells showed a stronger peptide-specific response (Fig. 2A). On the other hand, when CD3 and CD8 T cells from the same donor were stimulated with the D1-1 short peptide, CD3 T cells showed a far better peptide-specific response than CD8 T cells alone (Fig. 2B). This suggests that presence of CD4 T cells may help CD8 T cells. To confirm that the D1-1-specific response by CD3 T cells is not mediated by CD4 T cells, we also stimulated CD4 T cells with D1-1 and found no response could be generated (data not shown). These results confirmed our previous observation that CD3 T cells act as better effector cells than CD8 T cells alone in the induction of MHC class I peptide-specific response, most likely getting help from CD4 T cells (unpublished data).

### Cyclin D1-DR-1 peptide induced HLA-DR.B1-restricted cytotoxic CD4 T cells

**Figure 3:**
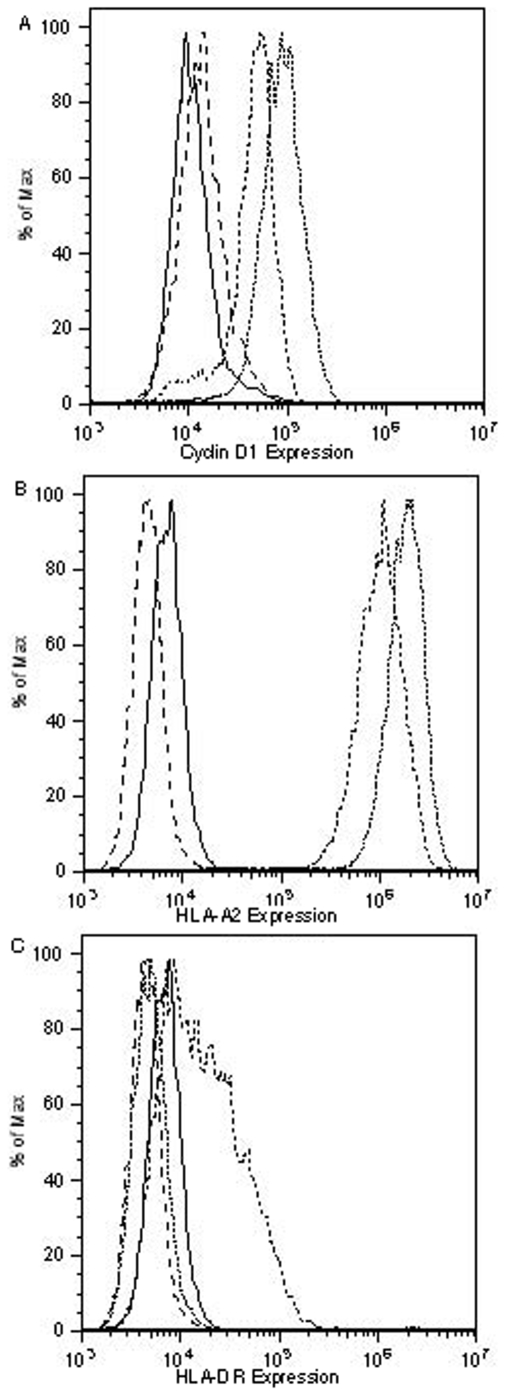
The expression of cyclin D1 and HLA molecules determined by flow cytometry. Cyclin D1 expression on mature DCs and MDA-MB-231 cell line was determined by intracellular protein staining using Cytofix/Cytoperm kit (A). The cells were stained with the mAb to human cyclin D1, or isotype control mouse IgG1, then were followed by the secondary antibody goat anti-mouse Ig (Fab)2 conjugated to FITC. Solid and dash lines are the DCs and MDA-MB-231 cells stained with isotype controls, respectively. The dash-dot-dash and dotted lines show the cyclin D1 expression on DCs and MDA-MB-231 cells, respectively. HLA-A0201 (B) or HLA-DR (C) expression on MBA-MB-231 was determined by surface staining of the cells with mAbs to HLA-A2 or HLA-DR, before and after treatment with IFN-γ. Dash and solid lines show the isotype controls before and after IFN-γ treatment, respectively. The dash-dot-dash and dot lines show HLA-A0201 expression before or after IFN-γ treatment (B). Similarly, the dash and solid lines show the isotype controls staining before and after IFN-γ treatment; the dot and dash-dot-dash lines show the HLA-DR expression before or after IFN-γ treatment (C).

**Figure 4:**
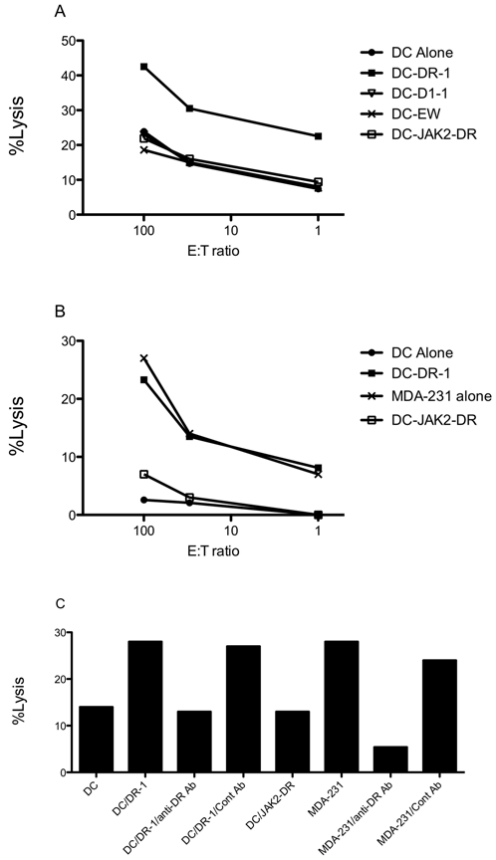
Induction of cytotoxicity by CD3 and CD4 T cells stimulated with DR-1 peptide. CD3 T cells from a HLA-A0201 donor were stimulated with DR-1 peptide for three times, and the cytotoxicity of the cells was measured by ^51^Cr-release assay against autologous DCs pulsed with or without indicated peptides (A). Similarly, the purified CD4 T cells from a HLA-A0201 and HLA-DR.B1-0701 donor was stimulated with DR-1 peptide and their cytotoxicity was measured by ^51^C-release assay against autologous DCs pulsed with or without indicated peptides, and MDA-MB-231 cell line (B). Antibody blocking was performed by pre-incubating target cells with anti-HLA-DR and isotype-matching irrelevant mAb (50 ug/ml) for 30 minutes before adding to the co-culture with effector cells at an E: T ratio of 100∶1 (C). The figure shows representative data from multiple similar experiments. All data points are the mean of triplicate microwell cultures.

Peptides that are presented on the surface of target cells bound to either HLA class I or class II molecules must first be processed either in the cytosol for class I peptides or in endocytic vesicles for class II peptides. Because the peptides derived from cyclin D1 were identified first by computer algorithms, it was necessary to determine whether these peptides were properly processed from the cyclin D1 protein and presented in the context of HLA molecules. Only the peptide fragments that are presented on the tumor cell surface can be recognized by peptide-specific T cells. We therefore tested if the DR-1 and D1-1 peptide-stimulated CD3 T cells were cytotoxic to tumor cells highly expressing cyclin D1. A HLA-A0201 positive human breast cancer cell line MDA-MB-231 was chosen because of its high expression of cyclin D1 [10]. We performed intracellular protein staining and confirmed the high level expression of the cyclin D1 compared to autologous mature DCs (Fig. 3A). This result is in agreement with reports from others that cyclin D1 is expressed at low level in normal tissues [27]. In addition, mature DCs show uptake of ^51^Cr, in contrast to resting lymphocytes, and therefore, could serve as a suitable target for ^51^Cr-release assay. The expression of HLA-A2 and DR was assessed by flow cytometry analysis followed by specific mAb staining. MDA-MB-231 cells express high levels of HLA-A2 molecule (Fig. 3B), which was slightly up-regulated by IFN-γ treatment. In contrast, the HLA-DR expression was mostly negative on MDA-MB-231 cells, but was significantly up-regulated by IFN-γ treatment (Fig. 3C). The IFN-γ pre-treatment did not alter the cyclin D1 expression on the MDA-MB-231 cells (data not shown) and therefore ruled out the possibility that the killing of the cells was due to the change of the cyclin D1 expression. CD3 T cells from different donors that were stimulated with DR-1, consistently killed the autologous DCs pulsed with DR-1peptide (10 out of 12 experiments), but not with the D1-1 peptide. Representative data from a donor (HLA-A0201+) is shown (Fig. 4A).

Furthermore, the CD3 T cells stimulated with D1-1 short peptide did not kill the DCs pulsed with the D1-1 short peptide in multiple donors with HLA-A0201 haplotype (data not shown). This was surprising, because the D1-1 peptide-stimulated CD3 T cells consistently responded to D-1- that was pulsed onto autologous APCs in IFN-γ ELISPOT assay. This suggested that DR-1-specific CD4 T cells might be the killer cells in the bulk CD3 T cells. To confirm if the DR-1-induced cytotoxicity was indeed mediated by CD4 T cells, we next used purified CD4 T cells as effectors. We found that the DR-1 stimulated CD4 T cells were also able to kill autologous DCs pulsed with DR-1 peptide, but not un-pulsed DCs or DCs pulsed with irrelevant DR1-binding peptide JAK2-DR. More importantly, the CD4 T cells also killed IFN-γ-pretreated MDA-MB-231 cell line, as shown in Fig. 4B (from a donor with HLA-A0201+ and HLA-DR.B1-0701+), but not the IFN-γ-untreated cells, indicating HLA-DR-mediated killing (data not shown). These experiments were repeated in six separate donors in nine experiments and were reproducible 7 times. To confirm the CD4 T cell responses are mediated via HLA- DR.B1, we next performed the blocking experiments using anti-HLA-DR mAb in ^51^CR-release assays. The mAb to HLA-DR significantly abrogated the DR-1 peptide-induced cytotoxicity of CD4 T cells against both DCs pulsed with DR-1 peptide and MDA-MB-231 cells (Fig. 4C). These results demonstrated that CD4 T cells recognized DR-1 epitope and are able to kill the cells expressing the epitope. On the contrary, D1-1 short peptide-stimulated CD3 or CD8 T cells did not kill MDA-MB-231, nor the other two HLA-A0201+ MCL cell lines, NCEB1 and Jeko, that also express high levels of cyclin D1 (data not shown). These results indicated, as suggested earlier, that the D1-1 epitope may not be processed in the target tumor cells, or CD8 T cells have a low-avidity for the epitope (Fig. 2B), which results in inefficient killing.

**Figure 5:**
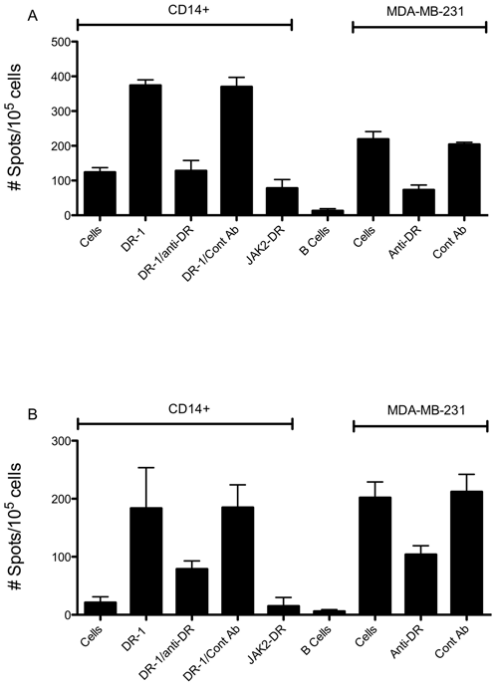
IFN-γ ELISPOT by CD4+CD25+ and CD4+CD25- T cells stimulated with cyclin D1-DR1 peptide. CD4+ (A) or CD4+CD25- T cells (B) from healthy donors were stimulated three times with DR-1 peptide, as described in the Materials and Methods. Stimulated T cells were challenged in IFN-γ ELISPOT assay with the stimulating peptide DR-1 or controls on CD14+ cells, B cells or MDA-MB-231 cells. Antibody blocking was performed by adding the anti-HLA-DR and isotype control mAb (50 ug/ml) in the cultures. The controls are: CD4 T cells cultures with un-pulsed CD14+ cells, irrelevant peptides JAK2-DR for HLA-DR.B1, and allogenic B cells from a donor who has matching HLA-DR.B1*0701 with MDA-MB-231 cells. The results represent the average spots in triplicate cultures +/− SD. The figures show one representative data from three similar experiments.

To investigate if depletion of T-regs could enhance the recognition of naturally processed epitopes in tumor cells by CD4 T cells, we compared DR-1-specific response between CD4+CD25+ T cells and CD4+CD25-T cells from the same donors in the IFN-γ ELISPOT assay. IFN-γ secretion between two populations were similar (Fig. 5). The mAb to HLA-DR abrogated the response to DR-1 peptide presented by autologous DCs and more importantly, the response to MDA-MB-231 cells. The results further supported the data from (Fig. 4C) that the DR-1 peptide can induce HLA-DR-restricted CD4 T cell response.

## Discussion

Increasing evidence has shown that vaccines with longer peptide sequences may perform better than minimal MHC class I binding peptide vaccines in anti-tumor immunity, in both mouse models and human trials [16], [18], [24]. Several mechanisms may contribute to improved efficacy. Inclusion of CD4 T cell epitopes in peptide vaccines help to induce and sustain effective CD8 T cell responses [28], [29]; longer peptides can be taken up and processed by DCs *in vivo* to induce effective CD8 T cell response, avoiding the induction of transient or tolerant CD8 responses if short peptides bind to less efficient APCs, such as B and T cells [30], [31]; there may be extra-cellular proteases known to trim peptides and therefore, short peptides may be rapidly biodegraded, while larger peptides are relatively protected and may benefit from additional extra-cellular processing [32], [33]. Finally, CD4 T cells, in some cases, themselves, may be cytotoxic to tumor cells.

In an attempt to improve vaccine efficacy, we designed long peptides from human cyclin D1-a protein that contain overlapping multiple epitopes of HLA class I and II epitopes. These peptides were intended to induce simultaneous CD4 and CD8 T cell responses, as shown for other combinations of T cell epitopes [25]. Importantly, this approach also allow us to measure if the CD8 epitopes are processed and presented. This is crucial for an effective vaccine therapy. In our initial screening of T cell response by IFN-γ ELISPOT assay, we found that four out of five long peptides we designed, based on the MHC-binding algorithms, induced strong CD3 T cell responses specific for the stimulating peptides. However, only one class II peptide, DR-1, induced CD3 T cells response against both DR-1 peptide and a short peptide D1-1, imbedded within the DR-1 sequence, suggesting that this CD4 epitope might be processed into the CD8 epitope. Although the other short peptides (D1-2 to D1-8) were predicted to be cleaved at C-terminal, none of them appeared to be processed, as assessed by recognition of these short peptides by CD3 T cells that have been stimulated with their respective long peptides. These results indicate that the CD8 epitopes predicted by MHC class I-binding and C-terminal cleavage algorithms are usually immunogenic, but not necessarily processed and presented appropriately. Therefore, our studies emphasize the importance of the verification of the function of the peptide sequences in vaccines, identified following epitope prediction algorithms, for processing and presentation by both host APCs and tumor cells.

To our surprise, CD3 T cells stimulated with DR-1 peptide not only induced IFN-γ secretion, but also killed the DCs pulsed with DR-1 peptide. In contrast, the D1-1 short peptide induced a strong peptide-specific response in IFN-γ ELISPOT assay, however, the peptide failed to induce cytotoxicity of either CD3 or CD8 T cells against peptide-pulsed autologous DCs and several tumor cells lines that highly express cyclin D-1. It is possible that although D1-1 peptide could activate CD8 T cells to produce IFN-γ, after three rounds of *in vitro* stimulations, they may have not differentiated into cytotoxic effector T cells. It is also possible that the frequency of the D1-1-specific CD8 T cells with high avidity for the epitope from bulk cultures may not be high enough, as shown in an analogous system by Stauss et al. [10]. In their study, only the high-avidity CTL clones, generated by limiting dilution, could kill the cyclin D1-expressing MDA-MB-231 cells, while the CTL clone with low-avidity failed to do so. In earlier studies (unpublished), cyclin D1 peptides 101–109 [10] and 228–236 [11] were identified because of their high binding avidity to HLA-A0201, predicted by computer algorithms. Peptide 228 was highly immunogenic measured by IFN-γ secretion, however, the peptides could not induce cytotoxicity in our system after three to four rounds of stimulation. Collectively, these studies suggest that the repetitive stimulations may be necessary to obtain high-avidity T cell clones *in vitro.*

Our studies support a strong helper CD4 T cell activity by showing a superior response of CD3 T cells than CD8 T cells against the same HLA class I peptide. We did not further investigate the mechanisms of this well-documented phenomenon. Rather, we focused on exploring a new possibility of inducing cytotoxic CD4 T cells directed at protein cyclin D1. To date, *in vivo* studies have shown that CD4 T cells can clear MHC class II-negative tumors via IFN-γ, which activates and induces tumoricidal macrophages. A recent study has provided a clear evidence that CD4 T cells can be more powerful anti-tumor effectors than CD 8 T cells, in a mouse model [34]. The authors suggested that interactions between CD4 T cells and NK cells or macrophages may have contributed to the anti-tumor immunity of the CD4 T cells. Moreover, Hunder et al. have reported that infusing NY-ESO-1-specific CD4 T cells, that were isolated and expanded *in vitro* from a patient with metastatic melanoma, induced complete tumor regression [35]. Although no definitive mechanisms are provided for the therapeutic efficacy of CD4 T cell in this case, the study demonstrated the important role of CD4 T cells in anti-tumor immunity in a patient with cancer. The direct cytotoxicity of CD4 T cells against tumor has not been firmly established, however, studies have shown that cyotoxic CD4 T cells can be induced that play an important role in clearance of pathogens, such as EBV and CMV infections [36], [37], [38]. In our studies, cyclin D1-DR1 peptide could prime both CD3 and CD4 T cells from donors with a broad range of HLA-DR.B1 haplotypes. More interestingly, the CD4 T cells from the HLA-DR.B1-matched donors are also able to kill the peptide-pulsed autologous DCs and more importantly, a tumor cell line, MDA-MB-231, expressing a high level of cyclin D1. The anti-HLA-DR antibody abrogated both the IFN-γ secretion and the cytotoxicity, confirming that the DR-1 peptide could induce a HLA-DR-mediated cytotoxic CD4 T cell response. Our studies provided the proof of concept that CD4 T cells can be cytotoxic against MHC class II positive tumor cells. This mechanism might be particularly important when tumor cells escape CD8 T cell attack by alteration of MHC class I expression; these “immune escape variants”, are seen commonly in tumor cells [39], Although some tumor cells do not express MHC class II molecules, IFN-γ secretion by T cells and NK cells in an inflammatory environment in tumor sites could up-regulate MHC class II expression and render tumor cells susceptible to CD4 T cell killing. Therefore, CD4-based treatment in vaccine strategy should be investigated further for developing more effective anti-tumor immunotherapy.

The impact of the CD4+CD25 T-regs in anti-tumor immunity has been widely reported in animal models [40], [41]. Although the effects of T-reg depletion remains to be determined in patients with cancers, recent *in vitro* studies have shown that the depletion of T-regs enhances vaccine-induced T cell immune responses in various cancers [42], [43], [44], [45]. Of particular interest is that the depletion of T-regs enhances the recognition of naturally processed tumor antigens, by eliminating the inhibition of T-regs on high-avidity T cells specific for tumor Ags, such as NY-ESO-1, in cancer patients [43], [44], [45]. We investigated the role of T-regs in the T cell response generated to the cyclin D1-derived peptides. No significant differences in CD4 T cell response against HLA-DR peptide DR-1 (Fig. 5), nor CD8 T cell responses against HLA-A2-specific peptide D1-1 (data not shown) were seen.

In conclusion, we identified a human cyclin D1-derived CD4 epitope potentially capable of interacting with multiple HLA-DR molecules, which provides priming and helper capacity in multiple donors. More importantly, the peptide DR-1 can induce cytotoxicity of CD4 T cells against tumor cells highly expressing cyclin D1 in the context of HLA-DR.B1 molecules. Considering the wide distribution of cyclin D1 in tumor cells, generation of specific CD4 CTLs, might add therapeutic activity in HLA-DR-expressing cancer cells.
